# Optimized Return to Sport Criteria Following the Bristow-Latarjet Procedure for Shoulder Stabilization: A Systematic Review and Meta-Analysis Protocol

**DOI:** 10.1055/s-0046-1824728

**Published:** 2026-07-28

**Authors:** Ewerton Borges de Souza Lima, Rafaelly Stavale, Paulo Henrique Schmidt Lara, Benno Ejnisman, Arun J. Ramappa, Alberto de Castro Pochini

**Affiliations:** 1 Sports Medicine and Physical Activity Course, Department of Orthopedics and Traumatology, Escola Paulista de Medicina, Universidade Federal de São Paulo, São Paulo, SP, Brazil; 2 Carl J. Shapiro Department of Orthopaedic Surgery, Beth Israel Deaconess Medical Center, Harvard Medical School, Boston, MA, United States.

**Keywords:** athletes, joint instability, return to sport, shoulder dislocation, systematic review as a topic, atletas, instabilidade articular, luxação do ombro, revisão sistemática como assunto, volta ao esporte

## Abstract

**Objective:**

To identify and synthesize return-to-sport (RTS) criteria reported after the Bristow–Latarjet procedure for anterior glenohumeral instability in athletes, and to evaluate the associated time to RTS, RTS rate, and instability recurrence, with the aim of informing an evidence-based combination of RTS criteria that may optimize earlier RTS while minimizing recurrence.

**Methods:**

This protocol follows the guidelines of the Preferred Reporting Items for Systematic review and Meta-Analysis-Protocols (PRISMA-P) statement and it has been registered in the International Prospective Register of Systematic Reviews (PROSPERO; CRD42024563618). We will include randomized and non-randomized comparative studies, cohort and case-control studies, and case series reporting explicit RTS criteria after Bristow–Latarjet in athletes, with a minimum follow-up of 12 months. Searches will be conducted in the PubMed, Web of Science, Embase, SciELO, CENTRAL and SPORTDiscus databases, as well as Google Scholar for grey literature, without restrictions on date, geography, or language. Two reviewers will independently screen and extract data using the Covidence (Veritas Health Innovation Ltd) software. The primary outcomes are time to RTS, RTS rate, and recurrence (using the time point closest to 12 months, when multiple assessments are available). The risk of bias will be assessed using the Risk of Bias 2 (RoB 2; the Cochrane Collaboration) for randomized clinical trials (RCTs), the Newcastle-Ottawa Scale (NOS) for non-randomized comparative studies, and the Joanna Briggs Institute (JBI) Critical Appraisal Checklist for Case Series, and the certainty of the evidence will be rated according to the Grading of Recommendations Assessment, Development, and Evaluation (GRADE) approach. When appropriate, random-effects meta-analysis and exploratory meta-regression will be performed; subgroup and sensitivity analyses are planned.

**Conclusion:**

The systematic review will provide a criterion-centered synthesis of RTS definitions and clearance strategies after Bristow–Latarjet, linking each criterion to clinically-relevant outcomes (time to RTS, RTS rate, and recurrence). The findings are expected to support more consistent, evidence-informed RTS decision-making and guide future prospective validation of optimized RTS criteria combinations.

## Resumo

**Objetivo:**

Identificar e sintetizar os critérios de retorno ao esporte relatados após o procedimento de Bristow-Latarjet para instabilidade glenoumeral anterior em atletas, e avaliar o tempo associado e a taxa de retorno ao esporte e a recorrência de instabilidade, com o objetivo de informar uma combinação baseada em evidências de critérios de retorno ao esporte que possa otimizar o retorno antecipado, para minimizar a recorrência.

**Métodos:**

Este protocolo segue as diretrizes da declaração Preferred Reporting Items for Systematic review and Meta-Analysis-Protocols (PRISMA-P) e está registrado no International Prospective Register of Systematic Reviews (PROSPERO; CRD42024563618). Incluiremos estudos comparativos randomizados e não randomizados, estudos de coorte e de caso-controle, e séries de casos que relatem critérios explícitos de retorno ao esporte após Bristow-Latarjet em atletas, com seguimento mínimo de 12 meses. As buscas serão realizadas nas bases de dados PubMed, Web of Science, Embase, SciELO, CENTRAL e SPORTDiscus, além do Google Acadêmico para a literatura cinzenta, sem restrições de data, geografia ou idioma. Dois revisores farão a triagem e a extração de dados de forma independente, usando o programa Covidence (Veritas Health Innovation Ltd). Os resultados primários são o tempo até o retorno ao esporte, a taxa de retorno ao esporte e a recorrência (usando o ponto de tempo mais próximo de 12 meses, quando várias avaliações estão disponíveis). O risco de viés será avaliado utilizando a ferramenta Risk of Bias 2 (RoB 2; the Cochrane Collaboration) para ensaios clínicos randomizados (ECRs), a Newcastle-Ottawa Scale (NOS) para estudos comparativos não randomizados, e a Lista de Verificação de Avaliação Crítica para Séries de Casos do Joanna Briggs Institute (JBI) para séries de casos, e a certeza da evidência será classificada por meio da abordagem Grading of Recommendations Assessment, Development, and Evaluation (GRADE). Quando apropriado, metanálise de efeitos aleatórios e metarregressão exploratória serão realizadas; análises de subgrupo e de sensibilidade estão planejadas.

**Conclusão:**

Esta revisão sistemática fornecerá uma síntese centrada nos critérios das definições de retorno ao esporte e das estratégias de liberação após Bristow-Latarjet relacionando cada critério a desfechos clinicamente relevantes (tempo até o retorno ao esporte taxa de retorno ao esporte e recorrência). Espera-se que os resultados apoiem a tomada de decisão de retorno ao esporte de forma mais consistente e baseada em evidências e orientem a futura validação prospectiva de combinações otimizadas de critérios de retorno ao esporte.

## Introduction

Anterior glenohumeral dislocation is common in young, physically-active individuals, 1 and it may progress to shoulder instability and recurrent dislocations, 2 limiting sports performance and quality of life. 3 Recurrence is frequent in high-risk athletes, which makes surgical stabilization a common recommendation. 4

The Bristow–Latarjet procedure is a bone-block technique widely used to treat anterior glenohumeral instability (AGHI), especially in the presence of glenoid bone loss, engaging Hill–Sachs lesions, failed prior soft-tissue stabilization, or in athletes at a high risk of recurrence, such as those participating in contact or overhead sports (activities that require athletes to repetitively lift and swing or throw their upper arm and shoulder in an arc above head height). 5 6 7

By restoring the anteroinferior glenoid arc and creating a dynamic sling effect through the conjoined tendon, Bristow–Latarjet provides reliable stability with recurrence rates of approximately 2 to 5%, and most athletes return to sport within 3 to 6 months. 5 6 7 8 However, surgical outcomes vary across studies mostly due to the differences in patient characteristics, rehabilitation protocols, and lack of standard return-to-sport (RTS) criteria to clear athletes for sports practice. 9

This heterogeneity is evident in the literature, with RTS timelines ranging from 6 weeks to 6 months. Protocols that enable earlier progression to sports practice are based solely on the known pathophysiology of bone graft healing time, with clearance sometimes considered as early as 6 to 8 weeks, 10 whereas the more conservative approach delays RTS until approximately 6 months based on the improvement plateau of the functional outcomes. 6 8 11 Such variation reflects uncertainty about what constitutes “safe readiness” and limits consistent counseling for athletes. 12

To date, there is no universal agreement on RTS readiness after Bristow–Latarjet. Published RTS criteria range from purely time-based clearance to multidomain approaches that incorporate clinical examination, range of motion, strength thresholds, functional testing, imaging confirmation, and patient-reported readiness. 13 Few studies have systematically mapped and compared these criteria, and even fewer have attempted to propose evidence-based combinations that balance a timely return with a low risk of recurrence. 13 14

As a result, the lack of standard RTS criteria affects the clinical practice, which may hinder a safe optimization of both time to RTS and postoperative stability. Therefore, the objective of the current protocol article is to describe the methodology for a systematic review that will identify and synthesize RTS criteria reported after the Bristow–Latarjet procedure and evaluate the associated outcomes. The review's ultimate goal is to provide an evidence-based criteria to support earlier RTS while minimizing recurrence rate.

## Study Type

The present study is a protocol for a systematic review that aims to provide a comprehensive analysis of the existing RTS criteria after the Bristow–Latarjet procedure for AGHI and to identify patterns and generate hypotheses on potentially-useful criteria combinations for optimized time to RTS and lower recurrence rates. It will follow the recommendations of the *Cochrane Handbook for Systematic Reviews of Interventions* 15 and the Preferred Reporting Items for Systematic Reviews and Meta-Analyses-Protocols (PRISMA-P) guidelines 16 ( Supplementary Material 1 ). The study has been registered in the International Prospective Register of Systematic Reviews (PROSPERO) under registration CRD42024563618.

## Review Question

The research question was formulated using the Population, Intervention, Comparison, Outcome, and Study Type (PICOS) framework: (P) athletes with AGHI undergoing the Bristow–Latarjet procedure; (I) RTS criteria applied after the Bristow–Latarjet procedure; (C) different RTS criteria; (O) time to RTS, RTS rate, and instability/dislocation recurrence; and (S) clinical studies reporting RTS criteria after Bristow–Latarjet. The inclusion and exclusion criteria are detailed in Table 1 .

**Table 1:** Inclusion and exclusion criteria

Criteria	Inclusion	Exclusion
**Study Type**	• Randomized controlled trials (RCTs); • Non-randomized controlled trials; • Prospective and retrospective cohort studies; • Case-control studies; and • Case series (n ≥ 5 and if quantitative data on the outcomes are presented)	• Case reports; • Technical notes, cadaver studies, biomechanical studies; • Systematic reviews, meta-analyses and other review types; and • Commentaries, letters to the editor, editorials, expert opinions, conference abstracts, posters and book chapters
**Population**	• Athletes of any age with primary or recurrent anterior glenohumeral instability (AGHI); and • Participants who underwent the Bristow-Latarjet procedure for the treatment of AGHI	• Patients with conditions other than AGHI (e.g., multidirectional instability, posterior instability); and • Patients who are not athletes or whose physical activity is not clearly defined
**Intervention**	• Return to sport criteria (time, muscle strength, range of motion, pain, radiographic evaluation etc.)	• Studies that do not clearly define the return to sport criteria
**Outcomes**	• Time until return to sport, return to sport rate, recurrence of injuries; and • Studies with a minimum follow-up of 12 months	• Studies without clear data on time until return to sport, return to sport rate, recurrence of injuries; and • Studies with a follow-up period shorter than 12 months

Studies including patients who practiced any sport before surgery will be eligible, regardless of competitive level. When available, the sporting profile described in each study will be extracted and categorized as recreational, competitive, or mixed/unclear. Definitions of RTS will also be recorded as reported by the original authors and categorized according to the outcome assessed, such as return to training, return to competition or return to preinjury level.

Non-randomized studies will be included, as the available evidence in this field is expected to consist predominantly of observational studies and case series. Restricting eligibility to randomized trials would likely exclude a substantial proportion of the evidence relevant to RTS and recurrence outcomes.

The systematic review intends to answer the following questions:

- What are the RTS criteria for the Bristow-Latarjet procedure identified in the literature?
- What are the time to RTS, RTS rate, and recurrence rate for each RTS criterion?
- What is the best combination of RTS criteria for lower recurrence rate and earlier RTS?

## Outcomes

The primary outcomes will be time to RTS, RTS rate, and recurrence of instability/dislocation after the Bristow–Latarjet procedure. For each included study, we will extract the RTS criteria and evaluate the respective outcomes.

To improve outcome comparability, recurrence-related outcomes will be defined a priori. Confirmed dislocation will refer to a documented episode of recurrent glenohumeral dislocation after RTS. Symptomatic subluxation will refer to a clinically reported or patient-reported symptomatic instability episode without confirmed complete dislocation. Reoperation will refer to any subsequent surgical procedure performed for recurrent instability or failure after the index procedure.

The secondary outcomes will include return to preinjury sport level, patient-reported outcome measures (Rowe, Western Ontario Shoulder Instability Index [WOSI], Single Assessment Numeric Evaluation [SANE], American Shoulder and Elbow Surgeons [ASES], or other validated scores), objective functional components embedded in RTS definitions (range of motion, strength testing, functional performance tests) and postoperative complications (infection, neurovascular injury, graft-related complications, stiffness, or reoperation for reasons other than recurrent instability). We will also summarize how RTS was defined and measured (return to training versus competition, sport-specific considerations).

When outcomes are reported at multiple follow-up time points, we will extract data from the assessment closest to 12 months postoperatively, provided that it occurs after the 12-month mark, for both primary and secondary analyses. Outcomes assessed before 12 months will not be considered for comparative analyses.

## Search Methods and Strategy

A comprehensive search will be conducted in the PubMed, Embase, Web of Science, SciELO, CENTRAL, and SPORTDiscus databases, with Google Scholar used for grey literature. PubMed and Embase were included for their broad biomedical indexing; Web of Science, for multidisciplinary coverage; SciELO, for regional literature (Latin American and Iberian journals); CENTRAL, for controlled studies; and SPORTDiscus, for sports medicine and rehabilitation literature relevant to RTS outcomes.

The search strategy ( Table 2 ) was intentionally focused on title/abstract terms to improve specificity and identify studies with a clear emphasis on the review topic. The selected terms incorporate relevant Medical Subject Headings (MeSH)/Embase Subject Headings (Emtree) concepts and related standardized terminology commonly used in the literature. These terms were iteratively tested during strategy development, and this approach also facilitates greater consistency across the different databases searched, thereby improving the reproducibility of the search strategy.

**Table 2:** Search strategies for each database

Database	Search strategy
**PubMed**	(shoulder instability[Title/Abstract] OR glenohumeral instability[Title/Abstract] OR glenohumeral dislocation[Title/Abstract] OR anterior glenohumeral instability[Title/Abstract] OR anterior shoulder instability[Title/Abstract]) AND (Latarjet[Title/Abstract] OR Bristow[Title/Abstract]) AND (return time[Title/Abstract] OR return sport*[Title/Abstract] OR return play[Title/Abstract] OR return practice[Title/Abstract]) AND (recurrence[Title/Abstract] OR recurrent[Title/Abstract] OR re-dislocation[Title/Abstract] OR re-injury[Title/Abstract] OR instability[Title/Abstract] OR apprehension[Title/Abstract] OR fail[Title/Abstract])
**Web of Science**	(shoulder instability OR glenohumeral instability OR glenohumeral dislocation OR anterior glenohumeral instability OR anterior shoulder instability) AND (Latarjet OR Bristow) AND (return time OR return sport* OR return play OR return practice) AND (recurrence OR recurrent OR re-dislocation OR re-injury OR instability OR apprehension OR fail)
**SciELO**	(“shoulder instability” OR “glenohumeral instability” OR “glenohumeral dislocation” OR “anterior glenohumeral instability” OR “anterior shoulder instability” OR “instabilidade do ombro” OR “instabilidade glenoumeral” OR “luxação glenoumeral” OR “instabilidade anterior do ombro” OR “instabilidade glenoumeral anterior” OR “inestabilidad de hombro” OR “inestabilidad glenohumeral” OR “luxación glenohumeral” OR “inestabilidad anterior de hombro” OR “inestabilidad glenohumeral anterior”) AND (Latarjet OR Bristow) AND (“return time” OR “return sport*” OR “return to sport*” OR “return to play” OR “return to practice” OR “retorno ao esporte” OR “retorno à prática esportiva” OR “retorno ao jogo” OR “tempo de retorno” OR “retorno al deporte” OR “retorno a la práctica deportiva” OR “regreso al deporte” OR “tiempo de retorno”) AND (recurrence OR recurrent OR “re-dislocation” OR reinjury OR “re-injury” OR instability OR apprehension OR failure OR recidiva OR recorrência OR reluxação OR “nova luxação” OR instabilidade OR “sinal de apreensão” OR falha OR recurrencia OR recidiva OR “re-luxación” OR “nueva luxación” OR inestabilidad OR “signo de aprensión” OR fallo)
**Embase**	(“shoulder instability”:ti,ab OR “glenohumeral instability”:ti,ab OR “glenohumeral dislocation”:ti,ab OR “anterior glenohumeral instability”:ti,ab OR “anterior shoulder instability”:ti,ab) AND (latarjet:ti,ab OR bristow:ti,ab) AND (“return time”:ti,ab OR “return to sport*”:ti,ab OR “return to play”:ti,ab OR “return to practice”:ti,ab) AND (recurrence:ti,ab OR recurrent:ti,ab OR “re-dislocation”:ti,ab OR “re-injury”:ti,ab OR instability:ti,ab OR apprehension:ti,ab OR fail*:ti,ab)
**CENTRAL**	((“shoulder instability” OR “glenohumeral instability” OR “glenohumeral dislocation” OR “anterior glenohumeral instability” OR “anterior shoulder instability”):ti,ab,kw) AND ((Latarjet OR Bristow):ti,ab,kw) AND ((“return time” OR “return sport*” OR “return play” OR “return practice”):ti,ab,kw) AND ((recurrence OR recurrent OR “re-dislocation” OR “re-injury” OR instability OR apprehension OR fail):ti,ab,kw)
**SPORTDiscus**	(TI (“shoulder instability” OR “glenohumeral instability” OR “glenohumeral dislocation” OR “anterior glenohumeral instability” OR “anterior shoulder instability”) OR AB (“shoulder instability” OR “glenohumeral instability” OR “glenohumeral dislocation” OR “anterior glenohumeral instability” OR “anterior shoulder instability”)) AND (TI (Latarjet OR Bristow) OR AB (Latarjet OR Bristow)) AND (TI (“return time” OR “return sport*” OR “return play” OR “return practice”) OR AB (“return time” OR “return sport*” OR “return play” OR “return practice”)) AND (TI (recurrence OR recurrent OR “re-dislocation” OR “re-injury” OR instability OR apprehension OR fail) OR AB (recurrence OR recurrent OR “re-dislocation” OR “re-injury” OR instability OR apprehension OR fail))
**Google Scholar**	(intitle:Latarjet OR intitle:Bristow) (“shoulder instability” OR “glenohumeral instability” OR “glenohumeral dislocation” OR “anterior glenohumeral instability” OR “anterior shoulder instability”) (“return to sport” OR “return to sports” OR “return to play” OR “return to practice” OR “return time”) (recurrence OR recurrent OR “re-dislocation” OR “reinjury” OR “re-injury” OR instability OR apprehension OR failure)

The search terms will cover concepts related to anterior shoulder/glenohumeral instability or dislocation, the Bristow–Latarjet procedure, RTS, and recurrence/instability outcomes. There will be no restrictions on date, geography, or language. If necessary, a translation tool will be used for data collection. An updated search will be carried out at the end of the study to ensure the inclusion of any recent studies.

Two independent reviewers will screen the titles and abstracts of identified articles for initial eligibility. Duplicate articles will be removed using the Covidence software (Veritas Health Innovation Ltd). Full-text articles of potentially-relevant studies will be assessed to determine eligibility based on inclusion and exclusion criteria. Discrepancies will be resolved by a third senior researcher.

To avoid double counting of participants, potentially-overlapping study populations will be assessed by comparing study center, recruitment period, author group, and sample characteristics. When overlap is suspected, the most comprehensive or most informative dataset will be included, and duplicate or substantially-overlapping cohorts will be excluded.

The reasons for exclusions will be documented in a table in the final evaluation. In the results, we will report the study selection process utilizing the PRISMA flow diagram of study selection.

## Data Extraction

As aforementioned, two reviewers will independently extract data using the Covidence software. The following information will be collected from each included study: study identification data, sample characteristics, intervention details, outcome measures, data on RTS, recurrence, and complications, risk of bias, and study quality. Data will be entered into a standardized extraction form ( Table 3 ) within Covidence and subsequently exported to Excel (Microsoft Corp.) for data management and analysis.

**Table 3:** Data extraction table with explanation of each item

Category	Details
**Study identification**	
Study identification	Unique identifier
Authors	All authors
Title	Complete title of the publication
Publication year	Year of publication
Journal name	Name of the journal
**Participant details**	
Age	Mean age and age range of participants
Gender	Number or percentage of male/female participants
Sample size	Total number of participants
Inclusion criteria	Specific criteria used for inclusion
Exclusion criteria	Specific criteria used for exclusion
Baseline characteristics	Detailed baseline characteristics (e.g., sport, previous injuries, competition level, symptom duration)
**Intervention details**	
Surgical technique	Bristow and/or Latarjet, open incision or arthroscopic, screws or button
Rehabilitation protocol	Information on postoperative rehabilitation protocols
**Outcome measures**	
Follow-up duration	Mean duration of the follow-up
Outcome measure endpoint	Time during follow-up in which outcomes were measured
Functional scores	Specific functional outcome scores (e.g., Constant-Murley, Western Ontario Shoulder Instability Index [WOSI], and American Shoulder and Elbow Surgeons [ASES] score)
**Data on return to sport**	
Definition of return to sport	How return to sport is defined (full participation, training only)
Time until return to sport	Mean time until return to sport
Level of return	Whether athletes returned to the same level of competition or a different level
Rate of return to sport	Rate of participants that were able to return to sport
**Recurrence and complications**	
Recurrence rate	Detailed data on recurrence rate and definition (endpoint closer to 12 months)
Complications	Any complications reported postsurgery (infection, graft failure)
**Risk of bias and quality assessment**	
Risk of bias assessment	Results from risk of bias tools (Risk of Bias 2 [ROB2] for randomized clinical trials, Newcastle-Ottawa Scale [NOS] for non-randomized clinical trials, and the Joanna Briggs Institute [JBI] Critical Appraisal Checklist for Case Series)
Quality score	Overall quality score or rating for each study
Level of evidence	Levels of Evidence of the Oxford Centre for Evidence-Based Medicine (OCEBM)

## Risk of Bias Assessment

Two reviewers will independently assess risk of bias for all included studies and will provide a written justification for each judgment; disagreements will be resolved by consensus or by consultation with a third senior reviewer. Randomized controlled trials (RCTs) will be evaluated using the Risk of Bias 2 (RoB 2; the Cochrane Collaboration) tool, non-randomized comparative studies will be assessed using the Newcastle-Ottawa Scale (NOS), and case series will be appraised with the Joanna Briggs Institute (JBI) Critical Appraisal Checklist for Case Series.

Publication bias will be assessed using funnel plots and Egger's test only when more than ten studies are available for a given meta-analysis, as these methods are not considered informative when the number of included studies is small.

## Quality of Evidence

The overall quality of evidence for each primary outcome will be assessed using the Grading of Recommendations Assessment, Development and Evaluation (GRADE) approach. 17 Evidence quality will be rated as high, moderate, low, or very low based on the domains of risk of bias, inconsistency, indirectness, imprecision, and publication bias. Randomized evidence will start at high certainty and may be rated down across these domains, whereas non-randomized evidence will start at low certainty and may be rated down or, when appropriate, rated up according to GRADE guidance. We will present GRADE judgments and key findings in a Summary of Findings table for the primary outcomes.

## Missing Data

When outcome data are missing, unclear, or incompletely reported, we will attempt to obtain the necessary information by contacting the corresponding authors. If data remain unavailable, we will report the extent and nature of missingness and include the study in the descriptive synthesis, while excluding it from any quantitative analysis that requires the missing information. When feasible, missing measures of dispersion will be derived from other available statistics using standard methods. We will not impute missing outcome values at the participant level; instead, analyses will be based on available data, and the potential impact of missing data will be explored through sensitivity analyses when appropriate.

## Subgroup Analysis

Subgroup analyses will be performed when sufficient data are available to explore potential sources of heterogeneity and clinically-relevant effect modifiers. Planned subgroups include type of RTS criterion, RTS definition, sport characteristics (collision/contact versus non-contact; overhead versus non-overhead), competitive level (recreational versus competitive) and surgical technique (open versus arthroscopic). When feasible, subgroup effects will be evaluated by comparing pooled estimates across subgroups and, if appropriate, by meta-regression; subgroup findings will be interpreted cautiously and considered exploratory.

## Sensitivity Analysis

Sensitivity analyses will be conducted to evaluate the robustness of the findings and the impact of key methodological decisions. When sufficient data are available, we will repeat meta-analyses after excluding studies at high risk of bias.

## Statistical Analysis

All statistical analyses will be based on aggregated data reported in the included studies. Data management and descriptive analyses will be conducted using the Covidence and Excel software. Meta-analyses and meta-regressions will be performed using statistical software such as Stata (StataCorp LLC) and/or R (R Foundation for Statistical Computing). Statistical significance will be set at a two-sided *p* -value < 0.05 for most analyses, except where otherwise specified ( *p* < 0.10 for tests of heterogeneity). All estimates will be presented with 95%CIs. Inter-rater agreement for study selection and risk-of-bias assessments will be quantified using Cohen's Kappa statistic with 95%CIs. For continuous outcomes, we will extract means and standard deviations. For dichotomous outcomes, we will calculate proportions with 95%CIs for each study or study group.

**Fig. 1:**
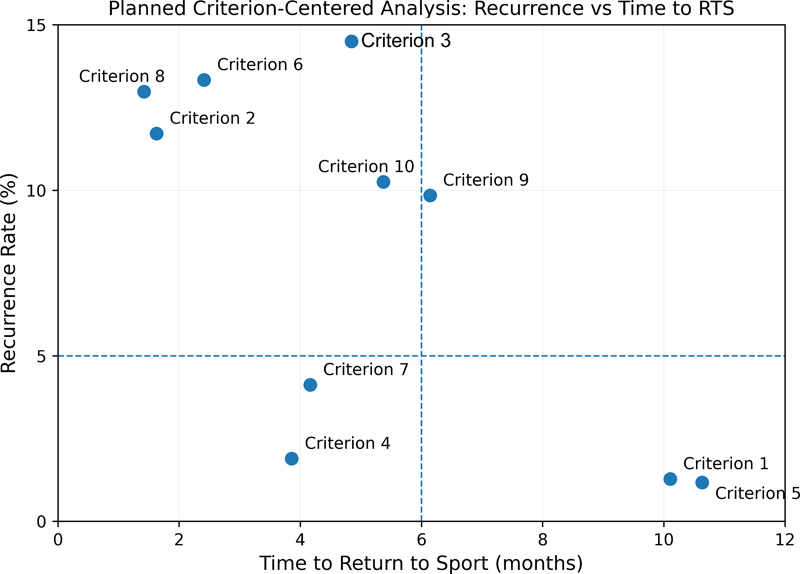
Conceptual example of the planned criterion-centered presentation of results. Each point represents a return-to-sport criterion (criteria 1–10), plotted according to time to RTS (months, x-axis) and recurrence rate (percentage, y-axis). Points closer to the bottom-left indicate earlier RTS and lower recurrence. This figure is illustrative only and does not represent preliminary results.

A narrative synthesis will initially be conducted to describe: the RTS criteria used in each study, the characteristics of the populations, surgical details, rehabilitation protocols, follow-up duration and risk of bias. Summary tables will present, for each included study, the RTS criteria, time to RTS, RTS rate, and recurrence rate. In addition, we will develop a scatter plot displaying the relationship between recurrence rate and time to RTS for each RTS criterion, enabling a clearer visual comparison and supporting the selection of the most clinically-useful criteria for practice ( Fig. 1 ).

Given that many eligible studies are expected to be case series or other single-arm designs, single-arm data will be included in the quantitative synthesis when appropriate. For proportion outcomes, such as RTS and recurrence rates, pooled estimates will be calculated using a random-effects meta-analysis of proportions with an appropriate variance-stabilizing transformation. Studies with rare events or zero-event data will not be excluded solely on this basis, and suitable statistical methods will be applied.

Quantitative pooling of time to RTS will be undertaken only when studies report sufficiently-homogeneous data in compatible formats. When these outcomes are reported as medians with interquartile ranges (IQRs) or ranges, they will be summarized descriptively and converted for pooling only when methodologically appropriate.

Meta-analysis will be performed when studies are considered sufficiently homogeneous in terms of population, intervention, and outcome definitions. Random-effects models will be used in view of the expected clinical and methodological heterogeneity. Statistical heterogeneity will be assessed using the Cochran Q test and quantified with the I ^2^ statistic, interpreted as low (∼ 25%), moderate (∼ 50%), or high (∼ 75%).

To explore whether different combinations of RTS criteria are associated with variation in time to RTS and recurrence rates, study-level meta-regression analyses may be performed. The primary predictor will be the category of RTS criteria used in each study, while additional covariates, including type of sport, competition level, surgical approach, follow-up duration, and overall risk of bias, may be considered depending on data availability. Any meta-regression will be considered exploratory and conducted only if feasible, with at least ten studies per covariate used as a rule of thumb to reduce the risk of overfitting and spurious findings.

## Discussion

Return-to-sport after the Bristow–Latarjet procedure is often presented as a key advantage of this technique, 18 19 yet the criteria used to clear athletes remain highly variable. This lack of standardization may contribute to both premature RTS with potential recurrence risk, as well as overly conservative delays that prolong time away from sport. 19

Researchers and orthopedic surgeons currently use a wide range of approaches to allow RTS, including time-based clearance, clinical milestones (pain, stability, range of motion), objective strength testing, functional performance measures, imaging confirmation of graft position/healing, and patient-reported readiness. 13 14 These criteria may be applied alone or combined in different ways. However, even when similar domains are considered, thresholds are rarely standardized, and RTS itself is inconsistently defined, such asreturn to training versus return to competition, or return at any level versus return to the preinjury level. 9 This variability creates important measurement problems, as athletes may be classified as “returned” under markedly-different standards, and recurrence may be reported using nonuniform definitions.

The lack of standard RTS criteria restricts the comparability across studies, increasing the risk of biased inferences and challenging clinical evidence-based decisions. Nevertheless, a systematic review is well suited to address this problem because it enables a structured mapping of how RTS criteria are defined in the literature and how they relate to outcomes across heterogeneous settings. Our protocol includes a rigorous methodology, which strengthens internal validity, but we anticipate practical challenges: incomplete reporting of RTS definitions, variability in follow-up schedules, inconsistent outcome terminology, and limited availability of comparable effect measures across studies. As a result, a quantitative synthesis may only be feasible for selected outcomes and subsets, and a narrative synthesis will remain essential to preserve clinical meaning.

The primary outcomes in the review were chosen because they reflect the core clinical trade-off after stabilization: enabling earlier return while maintaining durable stability. By linking each RTS criterion to these outcomes, the review aims to support a more explicit clinical rationale when selecting clearance strategies. We expect to find that time-based criteria alone are common and may be associated with wide variability in RTS timing and recurrence, whereas multidomain approaches may offer a more consistent safety profile. A key difficulty will be separating the effect of the RTS criterion itself from confounding factors such as sport type, competitive level, surgical technique, rehabilitation intensity, and baseline bone loss, all of which may influence clearance decisions and outcomes.

Several limitations are likely. The evidence base may be dominated by observational designs and case series, with variable risk of bias and incomplete reporting, which may reduce certainty and restrict meta-analysis. Differences in outcome definitions, follow-up time points, and the handling of recurrent events may further limit pooling and may require careful stratification and emphasis on narrative synthesis. Despite these challenges, the strength of the study lies in its criterion-centered approach and its focus on clinically-actionable outcomes. By synthesizing RTS criteria and their associated outcomes and grading the certainty of evidence, the review may help move RTS decisions from tradition toward clearer, evidence-informed standards, inform shared decision-making with athletes, and guide future prospective studies that test standardized, criterion-based RTS pathways after Bristow–Latarjet.
